# Analysis of family stigma and socioeconomic factors impact among caregivers of patients with early- and late-onset Alzheimer's disease and frontotemporal dementia

**DOI:** 10.1038/s41598-022-16400-2

**Published:** 2022-07-25

**Authors:** Lina Velilla, Natalia Acosta-Baena, Isabel Allen, Francisco Lopera, Joel Kramer

**Affiliations:** 1 Neuroscience Group of Antioquia, Medical School Antioquia University, Calle 62 N 52-59, 050010 Medellín, Antioquia Colombia; 2 Global Brain Health Institute, Memory and Aging Center University of California San Francisco, 1651 4th St Suite 212, San Francisco, CA 94143 USA; 3 Department of Epidemiology and Biostatistics, University of California San Francisco, San Francisco, CA USA

**Keywords:** Psychology, Diseases, Health care, Neurology, Risk factors

## Abstract

To the best of our knowledge, there are no research studies about socioeconomic factors, family stigma, and their psychological impact on early-onset dementia caregivers. We assessed the impact of family stigma and socioeconomic factors on psychological outcomes, quality of life (QoL), and caregiver burden among 150 caregivers of patients with early-onset Alzheimer’s disease due to E280A mutation in presenilin 1 (EOAD), frontotemporal dementia (FTD), and late-onset Alzheimer’s disease (LOAD). Caregivers of patients with EOAD presented a higher frequency of socioeconomic risk factors. Caregivers of FTD presented higher levels of family stigma and a higher prevalence of negative outcomes. We found family stigma to be a more suitable predictor of all outcomes. After adjusting for the type of dementia, dementia stage and behavioral changes, and caregiver age and education, family stigma was the most important factor associated with a higher risk of caregiver burden and a reduction in QoL in terms of energy fatigue and emotional wellbeing among early-onset dementia caregivers.

## Introduction

The evidence gathered on caregiver burden has been conducted primarily on caregivers of late-onset dementia. These studies have found that caregivers have a high risk of burden, anxiety, depression, and reduced QoL. The risk increases with socioeconomic factors such as low income, lack of community-based long-term care services, unemployment, and low education^1^. According to a few mixed qualitative and quantitative studies on late-onset dementia, family stigma has significant associations with increased caregiver burden, caregiving stress, social isolation, and decreased help^2,3^. These challenges are complicated by the particular circumstance of caring for loved individuals with early-onset Alzheimer’s disease or other early-onset dementias with a genetic component such as frontotemporal dementia (FTD) while at risk of developing the illness oneself. To the best of our knowledge, there are no research studies about socioeconomic factors, family stigma, and their impact on early-onset dementia caregivers. Hence, we aimed to fill this gap by assessing the effect that those factors have on caregiver burden, QoL, and emotional wellbeing in family caregivers of patients who belong to the largest worldwide cohort of EOAD due to the E280A mutation in presenilin 1 and patients with frontotemporal dementia (FTD) who are positive for other specific mutations.

Stigma has been understood as an adverse reaction and misinterpreted perception toward a negatively evaluated difference^4^, which has a deeply deleterious effect on the mental health of labeled individuals^5^. When individuals accept and internalize the stigma, they experience a misinterpretation and devaluation of themselves called self-stigma or internalized stigma^6,7^. On the other hand, when prejudices and discrimination are extended from stigmatized people to their friends or relatives who do not present marks of the stigmatized condition, it is known as courtesy stigma. When courtesy stigma is experienced by relatives or family caregivers and they internalized the process of self stigmatization it is known as family stigma^8^.

Family stigma is a significant and potentially modifiable contributor to caregiver burden, which remains scarcely addressed by quantitative and generalizable methods and represents a central issue for genetic populations at risk of developing EOAD^9^ or other forms of early-onset dementia with a genetic component such as FTD.

Thus, we hypothesize that socioeconomic conditions and familial stigma are associated with a higher risk of depressive symptoms, anxiety, lower quality of life, and caregiver burden among early-onset dementia caregivers.

The results of this research could contribute to designing tailored therapeutic strategies to reduce the caregiver burden in this unique population and to contribute to growing the body of knowledge about family stigma and dementia caregivers by addressing this issue with quantitative methods that allow us to objectively measure the risk of negative mental health outcomes for public health purposes.

## Materials and methods

### Design

This study is cross-sectional and observational with analytic outcomes and models. The aims were to determine whether caregivers of patients with EOAD had different socioeconomic features, affiliate stigma, and differences in caregiver outcomes relative to caregivers of FTD and LOAD patients and to analyze the associations between predictors such as socioeconomic and clinical factors, family stigma, and outcomes such as QoL, depressive symptoms, anxiety, and caregiver burden.

### Participants

The sample is made of non-dementia caregivers (asymptomatic) and the inclusion/exclusion criteria were designed for the caregivers. No caregiver had FTD, EOAD, or LOAD. All caregivers were clinically healthy. The participants were collected from the cohorts under follow-up by the Neuroscience Group of the University of Antioquia (GNA) Colombia. The study was approved by the bioethics committee of SIU (Sede de investigación Universitaria). All methods were performed under the relevant guidelines and regulations from the IRB. We included a sample size of 151 caregivers divided into 3 groups. Group number 1was made of 45 EOAD caregivers. Group number 2 comprised 51 FTD caregivers, and group number 3 comprised 55 LOAD caregivers. The inclusion criteria were as follows: (1) Being the main caregiver for at least one patient with a diagnosis of EOAD, FTD, or LOAD. (2) Availability to attend the GNA facility in Medellin for four hours to perform the study protocol. The exclusion criteria were having a psychiatric diagnosis of bipolar disorder or schizophrenia.

### Background testing

#### Factors and covariates

- *Socioeconomic factors (caregiver and patient questionaire)* This is a structured interview to identify basic demographic information and family features such as the number of relatives affected by dementia. This interview also explores the main socio-economic indicators, such as income, perceived income adequacy, education, labor conditions, housing, neighborhood safety, social support, health care access, health conditions current medical diagnoses, alcohol and tobacco consumption, activity level, and leisure activities. This interview asks about some general caregiving features as well as the number of hours caring and support to provide care.
- *Family stigma (caregiver test)* Affiliate Stigma Scale with caregivers of people with dementia^10^. This is a 22-item instrument-rated 4-point Likert scale. The scale assesses the three main domains described in the courtesy or association stigma model such as cognitive, made by 7 items in the scale; affect, with 7 items; and behavior, made of 8 items. Such domains describe the psychological aspects of family stigma which is defined in the stigma conceptualization as the process of self stigmatization in family members of patients with disabilities or degenerative conditions, The first domain assesses the caregiver´s cognitive attributions based on disease aspects such as the severity of the disease, the aesthetic appearance of the patient, and perceptions of dangerousness^11^. It includes questions such as “Others will discriminate against me if I am with my family member with dementia” or “My reputation is damaged because I have a family member with dementia at home”. The affect domain includes emotional reactions, both positive emotions (such as compassion and sorrow) and negative emotions (such as shame, embarrassment, and guilt)^11^. This domain has questions such as “I feel inferior because one of my family members has dementia” or “I feel emotionally disturbed because of my family member with dementia”. The third domain, behavioral, evaluate the caregiver´s responses such as avoidance or concealment. The score is made by calculating the total score and the mean score. The higher the score is, the greater the level of affiliate stigma.
- *Clinical stage (FAST) (Patient test)* Since the cognitive and functional deterioration that is implicit in the clinical course of dementia can affect the caregiver´s mood, quality of life, and wellbeing, we used the Functional Assessment Staging (FAST) to control that aspect of the patient/caregiver dyad. On this scale the highest the score the highest the decline and thus, the dementia stage. This measure allowed us to control in our statistical analysis all the caregiver´s outcomes accordingly to the severity and stage of the patient´s disease. The FAST assesses the de dementia evolution by considering seven stages from non-﻿dementia or asymptomatic (stage 1) to severe dementia (stage 7). The scale evaluates the integrated capacity underlying the cognitive function to undertake daily living. The Evaluation of changes in functional performance and activities of daily living skills is an essential aspect of the assessment of elderly individuals with dementia^12^.

- *Behavioral changes (Patient test)* The Frontal Behavioral Inventory (FBI)^13^ is a 24-item questionnaire that measures neuropsychiatrists symptoms and behavioral changes typical in FTD such as apathy, indifference, disorganization, inattention, personal neglect, aspontaneity, inflexibility, concreteness, loss of insight, logopenia, verbal apraxia, and alien hands. The higher the score, the higher the behavioral compromise.

#### Outcomes Instruments (caregivers tests)

- *Caregiver burden* The Zarit Burden Interview. We used the Colombian validation of this scale^14^. The validation study reported good reliability (total score alpha = 0.86). This test has 22 items on a five-point Likert type scale from 0 to 4, with higher scores indicating a higher burden. The scale measures the psychological burden and stress experienced by caregivers caring for people with dementia^15^.
- *Depressive symptomatology* CES-D (Center for Epidemiologic Studies Depression Scale)^16^. This test measures depressive symptoms by evaluating four domains; depressive mood, social aspects of depression, somatic symptoms, and positive affect. We used the Spanish version of the scale translated and validated for Hispanic speaker dementia caregivers. The scale is a five-point Likert type scale from 0 to 4. The higher the score the higher the level of depressive symptoms. a five-point Likert type scale from 0 to 4. The scale has been found reliable (Alpha > 0.85).
- *Anxiety* Spielberger State-Trait Personal Inventory^17^. We conducted the short form of the Spanish adaptation of the State-Trait Anxiety Inventory^18^. The scale is made of 40-items in a 4-point Likert type. The scale measures both trait anxiety (disposition to anxiety in different life situations) and state anxiety (anxiety at a particular moment) it consists of two separate sub-scales (STAI-T and STAI-S) each containing 20 items.
- *QoL* 36-Item Short Form Survey (SF-36). We used Spanish validation in a Colombian population^19^. In this validation, the inter-evaluator reliability was higher than 0,80, and the test-retest reliability was over 0,70. This test assesses the subjective perception of well-being within a socio-cultural context. The test includes 36 questions and 9 components of both physical and psychological health. The test provides both a general score and an individual component score. On this scale the highest the score the worse the perception of well-being.

### Statistical analysis

To examine associations between covariates, factors, and outcomes and to measure the direction and strength of each association, we performed bivariate and multivariate comparisons (ANOVA and chi-squared tests) and multivariate analysis of covariance (MANCOVA). Prior to MANCOVA, we confirmed that the dependent variables were continuous and linear, the dependent variable's normal distribution, homogeneity of regression slopes, and variance homogeneity. To estimate the risk (odds ratio) of having negative outcomes associated with family stigma, we performed four logistic regressions adjusting for patient clinical stage and behavioral change, caregiver age, and education. We confirmed assumptions for the independence of errors, linearity in the logit for continuous variables, absence of multicollinearity, and lack of strongly influential outliers. No corrections for multiple comparisons were made in this exploratory, observational study, and *p* < 0.05 was used as the criterion for statistical significance^20^.

### Ethical approval and informed consent

This study was approved and supervised by the Antioquia University Medical School Institutional Review Board under approval minute number 18-10-822. All participants signed the informed consent. This manuscript does not contain data from any individual person.

## Results

### Demographic and clinical features of the sample

Table 1 summarizes the baseline demographics and clinical features of the patients. There were significant differences in caregiver age and education. Caregivers of EOAD patients had the lowest scores on those variables. For clinical features, we found significant differences in behavioral changes and clinical stage. EOAD presented lower behavioral changes and clinical-stage evolution.

**Table 1:** Clinical and sociodemographic characteristics.

Variables	Group	*n*	Mean	Std. deviation	F	Sig
Caregiver's age	EOAD	45	46	11.4	11.5	0.000*
	FTD	51	55	11.6		
	LOAD	55	58	12.9		
Caregiver's years of education	EOAD	45	10.5	4.87	6.87	0.001*
	FTD	51	13.5	5.08		
	LOAD	55	13.8	4.38		
Caregiver's number of children	EOAD	45	1.87	1.47	0.64	0.527
	FTD	51	1.61	1.43		
	LOAD	55	1.56	1.34		
Patient's behavioral change (FBI)	EOAD	45	22.3	16	3.81	0.024*
	FTD	51	30.6	18.2		
	LOAD	55	30.4	15.5		
Patient's clinical stage (FAST)	EOAD	44	5.77	1.31	4.35	0.014*
	FTD	51	5.94	1.17		
	LOAD	55	5.29	1.07		

		EOAD	FTD	LOAD	CHI^2^	Sig
Caregiver Gender	Female	77.80%	90.20%	80.00%	3.06	0.217
	Male	22.00%	9.80%	20.00%		
Marital Status	Single	31.10%	13.70%	27.30%	5.03	0.540
	Married	57.80%	72.50%	58.20%		
	Widowed	6.70%	9.80%	9.10%		
	Divorced	4.40%	3.90%	5.50%		
	Full time	17.80%	13.70%	5.50%		
	Part time	6.70%	5.90%	5.50%		
Employment situation	Seasonal/non formal	17.80%	17.60%	30.90%	14.6	0.148
	Housewife	33.30%	41.20%	30.90%		
	Unemployed	8.90%	7.80%	0.00%		
	Retired	15.60%	13.70%	27.30%		
	Normal	55.60%	37.30%	41.80%		
Patient's behavior	Mild	6.70%	7.80%	10.90%	7.56	0.272
	Moderate	22.20%	17.60%	14.50%		
	Severe	15.60%	37.30%	32.70%		
*p < 0.05						

### Differences in QoL, caregiver burden, family stigma, depression, and anxiety traits among EOAD, FTD, and LOAD caregivers

The results for QoL of physical functioning, role limitations due to emotional problems, energy fatigue, emotional wellbeing, and general health indicate that the FTD group presented the worst performance in all those domains. The LOAD group presented the lowest score in caregiver burden and all domains of stigma, with significant differences. The EOAD group presented the highest scores for anxiety traits, and there were no significant differences in depression. Statistically significant differences between the three groups are seen in SF36 Physical functioning, Role limitations, Energy fatigue, Emotional wellbeing, and general health. All components of the Stigma scale showed significant differences between groups, and the Zarit burden score and STAI anxiety trait were significantly different between groups. The results of these analyses are shown in Supplemental Table 1.

### Socioeconomic characteristics among EOAD, FTD, and LOAD dementia caregivers

Table 2 shows differences in socioeconomic and sociodemographic caregiver features, such as having cared for other relatives with the same dementia in the past, depending on someone else providing care, the type of insurance, and housing.

**Table 2:** Socioeconomic characteristics among EOAD. FTD. and LOAD dementia caregivers.

		Group			Chi2	*P*-value
		EOAD	FTD	LOAD		
Cared for other relative with dementia in the past	Yes	29.5%	8.00%	21.8%	7.240	0.027*
	No	70.5%	92.0%	78.2%		
Who is helping to provide care	Relative	35.6%	38.0%	47.3%		
	Friend	0.00%	2.00%	0.00%	15.596	0.046*
	Paid caregiver	15.6%	32.0%	18.2%		
	No body	42.2%	14.0%	21.8%		
	More than one	6.67%	14.0%	12.7%		
Employment situation	Full time	17.8%	13.7%	5.45%	14.589	0.148
	Part time	6.67%	5.88%	5.45%		
	No formal (hourly)	17.8%	17.6%	30.9%		
	Housewife	33.3%	41.2%	30.9%		
	Unemployed	8.89%	7.84%	0.0%		
	Retired	15.6%	13.7%	27.3%		
Insurance company	None	0.00%	0.00%	1.82%	19.549	0.003*
	Sisben (Public)	17.8%	5.88%	3.64%		
	EPS (Taxable)	73.3%	60.8%	52.7%		
	Private insurance	8.89%	33.3%	41.8%		
Housing type	Family home	20.0%	29.4%	16.4%	12.561	0.014*
	Own house	48.9%	56.9%	74.5%		
	Leased	31.1%	13.7%	9.09%		
*p < 0.05						

### Multivariate models

We run a multivariate analysis of covariance (MANCOVA) to examine associations between covariates, factors, and outcomes and the direction and strength of each association, it means that we analyze what variables (sociodemographic factors, type of dementia or diagnostic group, behavioral changes, dementia stage, and family stigma) explained better caregiver´s outcomes such fatigue/energy for QoL, emotional wellbeing for QoL, caregiver burden and anxiety trait (Table 3). Before running the multivariate analysis of covariance (MANCOVA), we assessed assumptions for linear models. The dependent variables were continuous and linear and had normal distributions based on graphical inspection and statistical methods (Kolmogorov–Smirnov and Shapiro–Wilk tests). All variables are normally distributed and independent observations. There were no outliers. We explored linearity between the dependent-independent variables and found suitable patterns to move on to assess the homogeneity of regression slopes. There were no significant interactions.

**Table 3:** Parameter estimates for associations among outcomes and independent variables.

				IC 95%	
	B	SE B	*P*-value	Lower bound	Upper Bound
**QoL. SF36 Energy fatigue**					
Age	0.17	0.15	0.250	− 0.12	0.47
Education	0.28	0.42	0.509	− 0.55	1.11
Patient's clinical stage (FAST)	− 1.79	1.52	0.240	− 4.79	1.21
Blehavioral change (FBITotal)	− 0.13	0.10	0.190	− 0.33	0.07
Family stigma (Stigma scale total)	− 18.6	4.02	< 0.001*	− 26.5	− 10.6
DG (FTD)	− 7.24	15.1	0.633	− 37.1	22.6
DG (EOAD)	0.92	6.51	0.888	− 11.9	13.8
**QoL. SF36 Emotional wellbeing**					
Age	0.14	0.14	0.318	-0.14	0.42
Education	0.63	0.40	0.115	− 0.15	1.42
Patient's clinical stage (FAST)	− 1.77	1.44	0.220	− 4.61	1.07
Blehavioral change (FBITotal)	− 0.01	0.10	0.936	− 0.20	0.18
Family stigma (Stigma scale total)	− 20.2	3.80	< 0.001*	− 27.75	− 12.7
DG (FTD)	3.46	14.29	0.809	− 24.80	31.7
DG (EOAD)	− 2.00	6.16	0.746	− 14.2	10.2
**Caregiver burden: Zarit total**					
Age	− 0.14	0.08	0.105	− 0.31	0.03
Education	0.00	0.24	0.987	− 0.47	0.48
Patient's clinical stage (FAST)	0.85	0.86	0.329	− 0.86	2.55
Blehavioral change (FBITotal)	0.19	0.06	< 0.001*	0.08	0.31
Family stigma (Stigma scale total)	23.1	2.28	< 0.001*	18.6	27.6
DG (FTD)	12.4	8.59	0.152	− 4.63	29.4
DG (EOAD)	− 4.01	3.70	0.281	− 11.3	3.32
**Anxiety: STAI Anxiety trait**					
Age	− 0.02	0.04	0.547	− 0.10	0.05
Education	− 0.15	0.11	0.173	− 0.37	0.07
Patient's clinical stage (FAST)	− 0.04	0.39	0.928	− 0.82	0.74
Blehavioral change (FBITotal)	− 0.02	0.03	0.539	− 0.07	0.04
Family stigma (Stigma scale total)	5.60	1.04	< 0.001*	3.54	7.66
DG (FTD)	0.60	3.93	0.879	− 7.16	8.36
DG (EOAD)	1.85	1.69	0.275	− 1.49	5.19
*p < 0.005					

Using Wilks’ criterion, we found significant differences in family stigma (Wilk’s Λ = 0.52, F (4, 131)  = 30, *p* < 0.001), diagnostic group (Wilk’s Λ = 0.88, F (8, 262)  = 2.20, *p* = 0.028) and behavioral change FBI (Wilk’s Λ = 0.89, F (4, 131)  = 4.22, *p* = 0.003). No significant interaction was found. When performing the tests between-subject effects, we found significant effects between behavioral change and caregiver burden and family stigma and all outcomes, it means that family stigma was the only variable associated with the presence of lower energy, reduction in emotional wellbeing, higher caregiver burden and anxiety trait. Furthermore, behavioral changes were also able to explain an increase in caregiver burden. We analyzed the parameter estimates for associations among the outcomes, factors, and covariates (Table 3).

To measure the risk of negative psychological outcomes due to family stigma among caregivers we calculated the odds ratio (OR). For QoL (Energy/fatigue and emotional wellbeing), caregiver burden and anxiety trait. Our findings show that after controlling for age, education, clinical stage, and behavioral changes family stigma increased the risk for all outcomes except for anxiety. For Qol. (Energy/fatigue) we found that stigma explained 28% of the variance (OR 1.11, 95% IC: 1.06–1.17 P < 0,001). For Qol. (emotional wellbeing) stigma explained 28% of variance (OR 1.10, 95% IC: 1.05–1.15 *p* < 0,001). For Caregiver burden explained 46% of the variance (OR 1.23, 95% IC: 1.13–1.34 *p* < 0,001).

**Figure 1:**
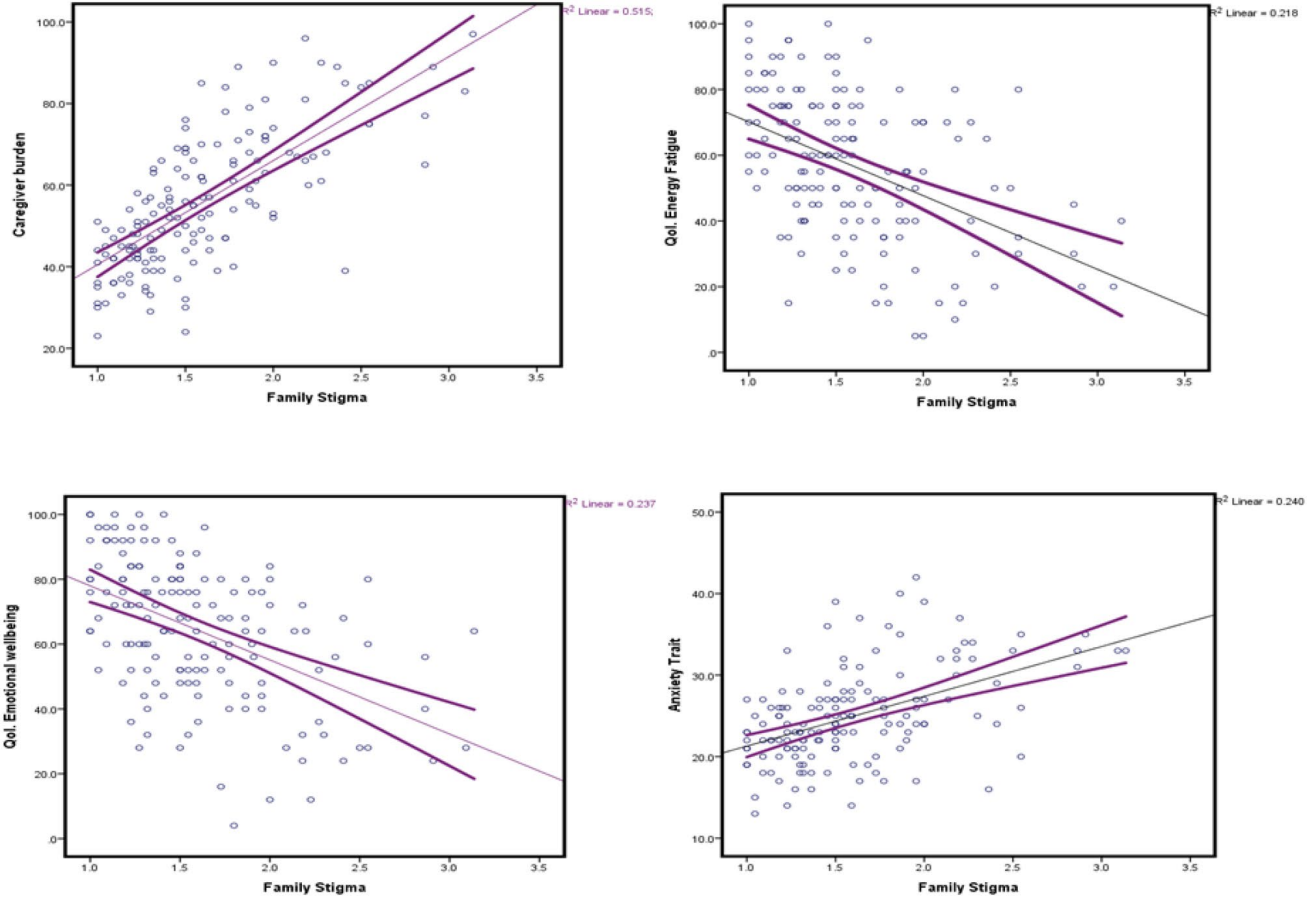
Negative psychological outcomes among caregivers according to family stigma.

We found significant associations between family stigma and energy/fatigue; the higher the score on the stigma scale was, the lower the performance in QoL. for suitable energy and low fatigue. The same pattern was observed for QoL. in the emotional wellbeing domain, where a unit increase in the stigma scale lowered the QoL performance by over 20 points. We found a modest association between behavioral change and an increase in caregiver burden. Similarly, a unit increase in the stigma scale produced a 23-point increase on the caregiver burden scale. In the anxiety trait, we found a significant association between stigma and anxiety, where a unit increase in the stigma scale score was 6.3 points higher than the score for the anxiety trait. Figure 1 shows the strong linear relationships between family stigma and the four outcomes.

## Discussion

Our study aimed to identify whether family stigma, patient clinical futures, and socioeconomic factors are associated with lower levels of QoL and a higher presence of depressive symptoms, anxiety, and caregiver burden among dementia caregivers. We also estimate the risk (OR) of presenting negative caregiver outcomes due to family stigma controlled by the caregiver’s age, education, patient clinical stage, and behavioral changes. To the best of our knowledge, this is the first study measuring with quantitative methods the risk of negative psychological outcomes due to family stigma and clinical and socioeconomic factors among dementia caregivers analyzed according to the type of dementia and including FTD, EOAD, and LOAD.

We selected to focus this study on early-onset dementia caregivers because they belong to genetic populations at risk of developing EOAD or other forms of early-onset dementia with a genetic component such as FTD. Both EOAD and FTD represent the two main causes of early-onset dementia^21^ and all of our cases are early-onset dementia caregivers.

Those populations with a family history of early-onset dementia have a high risk of marginalization and discrimination due to the dementia diagnosis, the clinical evolution experienced by the patients, and the challenges that caregiving for early-onset encounters^22^. This study contributes to understanding family stigma and caregiver outcomes with a different approach based on the specific type of dementia and the patient’s clinical features.

When analyzing socioeconomic features, we found that caregivers of EOAD were the youngest and those who had lower education. EOAD caregivers had a higher frequency of members who had taken care of a relative with dementia in the past, and they presented a higher frequency of not having help at all to provide care to their relative. On the other hand, FTD caregivers were the group that reported counting on paid support to provide care, and a higher frequency of caregivers with private insurance. The EOAD caregiver group relayed mostly on taxable and public insurance. The LOAD group was the group of caregivers with a higher frequency of members living in their own house, while FTD and EOAD lived mostly at the family house. The EOAD group presented with a higher frequency of potential socioeconomic factors; nevertheless, the FTD group had a higher frequency of negative psychological outcomes.

Our findings indicate that caregivers of patients with FTD exhibited the worst results in QoL and all the domains of family stigma. EOAD and FTD caregivers presented a higher caregiver burden, and FTD caregivers had higher levels of anxiety (trait). After adjusting for the patient’s clinical stage and behavioral changes, caregiver age, and education, we found that family stigma was the only predictor associated with worse outcomes. Family stigma decreased the levels of energy and increased fatigue. Similarly, we observed that when family stigma increased, emotional wellbeing was reduced. Stigma was associated with a higher caregiver burden. We estimated the risk of negative outcomes due to family stigma. We found that family stigma significantly increased the risk for all the outcomes associated, except for anxiety traits. After adjusting for potential confounders, family stigma was the variable more capable of explaining the variance in all outcomes assessed.

In summary, our bivariate results show that EOAD presented more socioeconomic aspects of risk for negative caregiver outcomes. Nonetheless, FTD caregivers exhibited a higher level of negative psychological and QoL outcomes and family stigma. When introducing adjustments in the multilinear model, family stigma remains the main factor explaining the variance in the outcomes and increasing the risk of negative psychological and reduced QoL outcomes, regardless of the patient's clinical stage and type of dementia.

Our results highlight the central role of family stigma in diminishing emotional wellbeing and QoL and increasing caregiver burden. These findings are in alignment with the study of Werner et al.^11^. They found that 50% of their sample of dementia family caregivers experienced self-stigma as a result of taking care of a relative with dementia AD-like and that the caregivers who experienced higher levels of family stigma presented the highest levels of caregiver burden. The authors confirmed that psychological factors were the main correlates of family stigma. In our study, family stigma was the most important factor associated with a higher risk of caregiver burden, reducing QoL in terms of energy fatigue and emotional wellbeing.

Another study reported findings similar to ours. The authors aimed to analyze how stigma and spiritual coping strategies mediated the associations between patient independence in daily life activities and anxiety, depression, caregiver burden, and the emotional components of QoL. For the case of stigma, they reported that it contributed to more than 40% of the variance in all outcomes, suggesting that the presence of stigma strengthened the harmful impact of the patient´s daily life independence impairment on each negative psychological and QoL outcome^23^.

Family stigma related to conditions such as dementia increases the burden among family caregivers because usually, they are the only ones in charge of managing the behavior of their relatives with dementia. This situation frequently leads to others' avoidance toward the caregiver and their family members with dementia^24^. Such social pressure due to social stigmatization could directly lead to a psychological disturbance among the caregiver. Similarly, isolation, scarce interaction with relatives and friends, embarrassment, and loss of social status are quite common situations and subjective feelings experienced by family caregivers who undergo family stigma^25^. Such a set of adverse psychosocial experiences has been found to negatively affect family members of persons with Alzheimer’s disease and is related to adverse consequences in a variety of diseases among the general population and for family caregivers^25^. Levy 2009 reported that stigma toward aging held in younger adulthood increased the risk of cardiovascular events, e.g., congestive heart failures, heart attacks, and strokes, over the next 38 years^26^.

Weisman de Mamani et al.^27^ found that caregivers with high levels of stigma presented more emotionally overinvolved (EOI) attitudes and behaviors that may lead the caregiver to be more intrusive, self-sacrificing, and being more prone to be critical or intrusive toward their relative in an attempt to control symptomatic behaviors. Furthermore, they found that high EOI is a potential mediator between family stigma and the psychological factors of the QoL. This finding could be explained because high EOI is linked to lower mental health in caregivers themselves^28^. The author's findings suggested that targeting family stigma in psychotherapy could diminish levels of high EOI and indirectly increase caregiver QoL.

The relationship between family stigma and negative psychological outcomes among early-onset dementia caregivers is particularly relevant in the Latin American context due to sociocultural patterns and financial constraints that could impose severe pressure on the caregivers to provide care for their relatives. In previous research that examined the impact of family stigma among caregivers of patients with schizophrenia, it was found that the negative impact of this form of stigma is particularly marked in settings where family cohesion is high, such as happens to cultural patterns in Latinamerica. In many low- and middle-income countries, most people living with brain/mental health diseases live with their families, and they are dependent on them for both economic support and everyday care; hence, the caregivers end up displaying several of the roles occupied by health or social care staff in high-income country settings. This study's authors reported that stigma was independently associated with higher levels of positive symptoms of schizophrenia, higher levels of disability, younger patient age, and household education at the secondary school level^29^. These results emphasize the association between family stigma and socioeconomic, demographic, and disease clinical factors and highlight the need to grow the body of knowledge about this regard in low- to middle-income countries, where caregivers are potentially more exposed to cultural and financial constraints that could represent a higher risk of family stigma and negative psychological outcomes.

Following those findings of the impact of sociodemographic factors on stigma and caregiver wellbeing and burden, we found in our study that most of the caregivers were females. In a previous study, the Alzheimer Association showed that two-thirds of caregivers were women where one-third of whom are daughters^30^. This characteristic of dementia care is very common in Latin American and other low /middle-income countries^31^ and, needs to be highlighted and addressed from multiple perspectives due to the impact that it may have on gender equity and women's access to higher levels of education, income and their role in society. In a previous study was reported that compared to sons, daughters caregivers of AD patientsperformed more caregiving tasks, suffered more guilt about the care they were able to provide, were less likely to accept support from their partners, and were more susceptible to abandon the labor market to care for their parent^32^.

The Family Caregiver Alliance in 2013^33^ reported that women remain to be the most frequent care provider for dementia relatives; they are who manage the more difficult caregiving tasks and they are the most affected by the consequences of caregiver burden such as an increase in depression, fatigue, anxiety and cardiovascular diseases^34^. As it is often common in all Latino and low/middle-income countries, in our study females were the main provider of dementia care among all groups. When we evaluated according to dementia type, we did not find differences by gender. Thus, we need to call attention to this finding.

Likewise, another sociodemographic factor that should be addressed further in the future is the dyad caregiver/patient age. Both FTD and EOAD as early-onset caregivers are likely in the same age group as their relatives with the disease. This is an important factor that should be addressed further to understand how it can affect psychological outcomes among caregivers.

## Conclusions

In conclusion, our findings suggested that even after adjusting for the type of dementia, dementia stage and behavioral changes, and caregiver age and education, family stigma was the most important factor associated with a higher risk of caregiver burden and a reduction in QoL in terms of energy fatigue and emotional wellbeing among early-onset dementia caregivers. These results indicated the importance of tackling family stigma, both socially with stigma awareness campaigns and individually in therapeutic measures. All of these factors improve caregiver health, psychological wellbeing, and QoL.

### Limitations

This study has multiple strengths since it was carried out in populations under follow-up by medical and psychological personnel who are experts in dementia of the GNA, with continuous measurements of the patient. For this study, however, the caregiver surveys were carried out in a cross-sectional manner without tracking over time. The population included were caregivers who lived in Medellin, an urban area. The rural population was not included, and the sociodemographic aspects may be different from those found in this sample. It is unknown whether the outcomes may also be different. The GNA carries out social strategies permanently aimed at improving the well-being of caregivers, mainly in the population with EOAD and LOAD risk. The population with FTD may be less involved in interventions. However, the type of dementia does not appear to be related to the outcomes evaluated. New studies are needed in similar populations, covering both the caregiver and the patient together to confirm the role of stigma in Latin America and determine the possibilities of better intervention.

## Supplementary Information

Supplementary Information.
