# Supplementary material for: Analysis of family stigma and socioeconomic factors impact among caregivers of patients with early- and late-onset Alzheimer's disease and frontotemporal dementia
Source: Sci Rep. 2022 Jul 25;12:12663. doi: 10.1038/s41598-022-16400-2 (PMC9314345; doi:10.1038/s41598-022-16400-2)
Supplement: Supplementary file 1 — Supplementary Information. [file 41598_2022_16400_MOESM1_ESM.docx]

| **APPENDIX**  **Supplemental Table 1. Differences in Qol. caregiver burden, stigma, depression and anxiety traits among EOAD, FTD and LOAD caregivers** | | | | | | | | |
| --- | --- | --- | --- | --- | --- | --- | --- | --- |
|  | | n | Mean | Std. Deviation | 95% Confidence Interval for Mean | |  |  |
|  |  |  |  |  | Lower Bound | Upper Bound | F | Sig. |
| SF36 Physical functioning | EOAD | 45 | 80,7 | 19,0 | 75,0 | 86,4 | 4,636 | 0,011* |
|  | FTD | 51 | 70,3 | 24,3 | 63,5 | 77,1 |  |  |
|  | LOAD | 55 | 81,7 | 19,1 | 76,6 | 86,9 |  |  |
| SF36 Role limitations due to physical health | EOAD | 45 | 59,4 | 44,4 | 46,1 | 72,8 | 0,810 | 0,444 |
|  | FTD | 51 | 55,4 | 47,3 | 42,1 | 68,7 |  |  |
|  | LOAD | 55 | 66,9 | 49,7 | 53,5 | 80,3 |  |  |
| SF36 Role limitations due to emotional problems | EOAD | 45 | 58,5 | 43,3 | 45,5 | 71,5 | 3,230 | 0,042* |
|  | FTD | 51 | 47,1 | 44,8 | 34,5 | 59,7 |  |  |
|  | LOAD | 55 | 67,9 | 38,5 | 57,5 | 78,3 |  |  |
| SF36Energyfatigue | EOAD | 45 | 58,8 | 21,0 | 52,5 | 65,1 | 3,070 | 0,049* |
|  | FTD | 51 | 50,7 | 21,2 | 44,7 | 56,7 |  |  |
|  | LOAD | 55 | 60,7 | 23,0 | 54,5 | 67,0 |  |  |
| SF36 Emotional wellbeing | EOAD | 45 | 69,9 | 19,7 | 63,9 | 75,8 | 4,848 | 0,009* |
|  | FTD | 51 | 57,1 | 23,2 | 50,6 | 63,6 |  |  |
|  | LOAD | 55 | 66,5 | 20,1 | 61,1 | 72,0 |  |  |
| SF36 Social functioning | EOAD | 36 | 70,0 | 28,3 | 61,5 | 78,5 | 1,754 | 0,177 |
|  | FTD | 41 | 61,5 | 30,2 | 53,0 | 70,0 |  |  |
|  | LOAD | 49 | 71,6 | 29,1 | 63,7 | 79,5 |  |  |
| SF36 Pain | EOAD | 45 | 68,3 | 27,0 | 60,2 | 76,4 | 2,872 | 0,060 |
|  | FTD | 51 | 58,2 | 29,1 | 50,1 | 66,5 |  |  |
|  | LOAD | 55 | 70,0 | 24,1 | 63,4 | 76,5 |  |  |
| SF36 General health | EOAD | 45 | 62,0 | 26,5 | 54,0 | 70,0 | 3,809 | 0,024* |
|  | FTD | 51 | 56,0 | 29,7 | 47,6 | 64,3 |  |  |
|  | LOAD | 55 | 70,5 | 25,6 | 63,6 | 77,5 |  |  |
| SF36 Health change | EOAD | 45 | 47,8 | 26,6 | 39,8 | 55,8 | 1,028 | 0,360 |
|  | FTD | 51 | 43,1 | 23,5 | 36,5 | 49,7 |  |  |
|  | LOAD | 55 | 50,0 | 25,0 | 43,2 | 56,8 |  |  |
| Zarit total | EOAD | 45 | 58,9 | 17,2 | 53,7 | 64,1 | 3,707 | 0,026* |
|  | FTD | 51 | 58,0 | 17,6 | 53,0 | 62,9 |  |  |
|  | LOAD | 55 | 51,0 | 13,4 | 47,4 | 54,7 |  |  |
| Stigma scale total | EOAD | 45 | 36,6 | 11,5 | 33,1 | 40,0 | 6,472 | 0,002* |
|  | FTD | 51 | 38,0 | 10,3 | 35,1 | 40,9 |  |  |
|  | LOAD | 55 | 31,5 | 7,38 | 29,5 | 33,5 |  |  |
| Stigmas scale average | EOAD | 45 | 1,7 | 0,52 | 1,51 | 1,82 | 6,056 | 0,002* |
|  | FTD | 51 | 1,7 | 0,48 | 1,58 | 1,85 |  |  |
|  | LOAD | 55 | 1,4 | 0,33 | 1,34 | 1,52 |  |  |
| Stigma scale emotional component | EOAD | 45 | 13,5 | 5,06 | 11,9 | 15,0 | 4,934 | 0,008* |
|  | FTD | 51 | 14,5 | 4,32 | 13,3 | 15,7 |  |  |
|  | LOAD | 55 | 11,8 | 3,84 | 10,8 | 12,9 |  |  |
| Stigma scale emotional component average | EOAD | 45 | 1,92 | 0,72 | 1,70 | 2,13 | 4,623 | 0,011* |
|  | FTD | 51 | 2,05 | 0,62 | 1,88 | 2,23 |  |  |
|  | LOAD | 55 | 1,69 | 0,55 | 1,54 | 1,84 |  |  |
| Stigma scale behavioral component | EOAD | 45 | 12,2 | 4,15 | 10,9 | 13,4 | 3,764 | 0,025* |
|  | FTD | 51 | 12,4 | 4,44 | 11,2 | 13,7 |  |  |
|  | LOAD | 55 | 10,5 | 2,83 | 9,78 | 11,3 |  |  |
| Stigma scale behavioral component average | EOAD | 45 | 1,52 | 0,52 | 1,36 | 1,68 | 3,667 | 0,028* |
|  | FTD | 51 | 1,55 | 0,55 | 1,39 | 1,70 |  |  |
|  | LOAD | 55 | 1,32 | 0,35 | 1,22 | 1,41 |  |  |
| Stigma scale cognitive component | EOAD | 45 | 10,9 | 3,52 | 9,85 | 12,0 | 7,085 | 0,001* |
|  | FTD | 51 | 11,1 | 3,13 | 10,3 | 12,0 |  |  |
|  | LOAD | 55 | 9,18 | 2,05 | 8,63 | 9,74 |  |  |
| Stigma scale cognitive component average | EOAD | 45 | 1,55 | 0,50 | 1,40 | 1,70 | 6,502 | 0,002* |
|  | FTD | 51 | 1,58 | 0,45 | 1,45 | 1,70 |  |  |
|  | LOAD | 55 | 1,31 | 0,29 | 1,23 | 1,39 |  |  |
| CESD total | EOAD | 45 | 13,4 | 14,3 | 9,16 | 17,7 | 2,053 | 0,131 |
|  | FTD | 51 | 15,5 | 15,5 | 11,2 | 19,9 |  |  |
|  | LOAD | 55 | 10,2 | 11,3 | 7,13 | 13,2 |  |  |
| CESD depressive mood | EOAD | 45 | 5,09 | 6,41 | 3,16 | 7,01 | 2,113 | 0,124 |
|  | FTD | 51 | 5,75 | 6,59 | 3,89 | 7,60 |  |  |
|  | LOAD | 55 | 3,45 | 4,71 | 2,18 | 4,73 |  |  |
| CESD social aspects | EOAD | 45 | 1,38 | 1,75 | 0,85 | 1,90 | 0,937 | 0,394 |
|  | FTD | 51 | 1,86 | 2,00 | 1,30 | 2,43 |  |  |
|  | LOAD | 55 | 1,51 | 1,69 | 1,05 | 1,97 |  |  |
| CESD somatic symptoms | EOAD | 45 | 3,93 | 3,70 | 2,82 | 5,04 | 1,739 | 0,179 |
|  | FTD | 51 | 4,39 | 4,16 | 3,22 | 5,56 |  |  |
|  | LOAD | 55 | 3,07 | 3,23 | 2,20 | 3,94 |  |  |
| CESD positive affect | EOAD | 45 | 3,04 | 4,01 | 1,84 | 4,25 | 1,835 | 0,163 |
|  | FTD | 51 | 3,53 | 4,23 | 2,34 | 4,72 |  |  |
|  | LOAD | 55 | 2,15 | 3,07 | 1,32 | 2,98 |  |  |
| STAI anxiety state | EOAD | 43 | 24,3 | 4,78 | 22,8 | 25,7 | 1,087 | 0,339 |
|  | FTD | 51 | 25,2 | 4,11 | 24,0 | 26,3 |  |  |
|  | LOAD | 55 | 24,0 | 4,13 | 22,9 | 25,1 |  |  |
| STAI anxiety trait | EOAD | 43 | 25,8 | 5,51 | 24,1 | 27,5 | 4,507 | 0,013* |
|  | FTD | 51 | 26,2 | 6,49 | 24,4 | 28,0 |  |  |
|  | LOAD | 55 | 23,2 | 4,75 | 21,9 | 24,5 |  |  |
| *p<0,05 |  |  |  |  |  |  |  |  |
